# Integrative Multimodal Profiling of TAp73 and DNp73 Reveals Isoform-Specific Transcriptomic Coregulator Landscapes in Cancer Programs

**DOI:** 10.3390/biom16010063

**Published:** 2025-12-31

**Authors:** Steffen Möller, Alf Spitschak, Nico Murr, Brigitte M. Pützer

**Affiliations:** 1 Institute of Experimental Gene Therapy and Cancer Research, Rostock University Medical Center, 18057 Rostock, Germany; steffen.moeller@uni-rostock.de (S.M.); alf.spitschak@med.uni-rostock.de (A.S.);; 2 Department of Life, Light and Matter, University of Rostock, 18055 Rostock, Germany

**Keywords:** p73, coregulatory transcription factors, CUT&RUN chromatin profiling, cofactor enrichment analysis, EMT, cancer patient signature

## Abstract

(1) Background: The transcription factor p73 exists in multiple isoforms with divergent functions in cancer. While DNp73 promotes stemness, epithelial–mesenchymal transition (EMT), and metastasis, the tumor-suppressive isoform TAp73 can also switch to promoting cancer progression. How isoforms sharing the same DNA-binding domain produce divergent outcomes remains unclear. (2) Methods: Here, we performed CUT&RUN in combination with JASPAR, transcriptomics, proteomics, patient survival and gene expression data to map genome-wide and promoter-associated DNA-binding and coregulatory transcription factor (coTF) profiles of TAp73α and DNp73β in melanoma cells. (3) Results: Systematic screening for motif enrichment in cancer hallmark gene sets revealed TAp73- and DNp73-specific coTF repertoires with distinct functions. We identified a coregulator signature for EMT genes enriched for both isoforms that has tumor context-dependent effects on survival and correlates with unfavorable patient prognosis. Of these EMT-associated coTFs, PATZ1 was validated as a novel direct interactor of DNp73β. (4) Conclusions: Our results provide a comprehensive reference map of p73 isoform-specific binding and coregulator recruitment and establish a workflow to model their influence on cancer reprogramming with implications for AI-based individualized therapy.

## 1. Introduction

The transcription factor p73 belongs to the p53 tumor suppressor family. While p53 is firmly established as the “guardian of the genome,” p73 displays broader regulatory versatility through numerous isoforms. These fall into two main groups: TA isoforms retain the N-terminal transactivation domain, whereas DN isoforms lack it due to alternative promoter usage (ΔN) or splicing (ΔEx2p73, ΔEx2/3p73, and ΔN′p73). Alternative splicing also modifies the C terminus. The full-length p73α contains the SAM domain, which is absent in the seven shorter variants (β–θ) [1,2]. All isoforms share the same DNA-binding domain (DBD), which drives competition for the same promoter sites and consequently alters transcriptional programs. Functionally, TAp73 promotes apoptosis and compensates for p53 loss, therefore acting as a tumor suppressor. In contrast, DNp73 is predominantly antiapoptotic and oncogenic [3], suppressing TAp73 and p53 activity by competing for DNA binding or forming inactive hetero-oligomers [4].

The present study focuses on two clinically well-characterized p73 isoforms, TAp73α and DNp73β (ΔEx2/3p73β). Their relative abundance impacts whether a tumor cell undergoes apoptosis or activates cancer programs. A shift toward DNp73 promotes metastasis initiation and therapy resistance, whereas restoring TAp73 activity could offer therapeutic potential. However, recent findings challenge this paradigm and reveal a greater regulatory complexity, particularly regarding the role of TAp73 in cancer progression. Beyond its tumor-suppressive functions, TAp73 can also foster oncogenic processes, including antiapoptotic signaling and angiogenesis [2,5]. We recently reported that TAp73 is converted from a suppressor to an oncogenic transcription factor through the interaction of its SAM domain with the HDAC2/REST repressor complex, thereby activating GABBR2-mediated cancer-promoting functions such as EMT and invasiveness [5]. These findings clearly demonstrate how transcriptional context-dependent protein–protein interactions (PPIs) with coregulatory factors govern p73 activity. Several mechanistic models have been proposed: Modulation by YAP1 or PIN fine-tunes the response to DNA damage by binding (tethering) to partners, such as NF-Y, that enable promoter occupancy in the absence of canonical p73 response elements (p73-REs), and composite regulation via adjacent motifs supports cooperative activation, as exemplified by TAp73β and Sp1 at the PUMA promoter [2]. These mechanisms underscore that the promoter architecture directly shapes PPIs to determine p73 transcriptional programs. The dual role of the supposedly tumor-suppressive isoform TAp73α as a procancer factor has been described in detail elsewhere [2]. Regardless of these individual studies, isoform-specific chromatin binding and interactions with coregulators have not been comprehensively characterized genome-wide. Previous chromatin-immunoprecipitation (ChIP)-seq approaches in cancer cells revealed a large number of p73 target genes and showed that single-isoform analyses are feasible [6,7]. However, ChIP-seq itself is limited by high background, large input requirements, and relatively low resolution, which can obscure subtle isoform-specific events. In this regard, newer techniques such as CUT&RUN overcome these restrictions through higher precision and improved signal-to-noise ratios. Yet experimental assays or large datasets alone cannot fully resolve the intricacy of transcriptional regulation. Integrative strategies are essential, as single-layer analyses at the gene or protein level fail to capture the regulatory complexity of cancer.

To address these challenges, we established an integrative workflow that combines CUT&RUN assays with JASPAR motif predictions, transcriptomics, proteomics, and patient data to create a p73 binding map, uncover isoform-specific coregulators, and provide a framework for AI-based models for cancer stratification and therapy guidance.

## 2. Materials and Methods

### 2.1. Cell Culture, Viral Vectors and Treatments

Human SK-Mel-29 and SaOS-2 cell lines were maintained at 37 °C in a humidified atmosphere with 5% CO_2_ in Dulbecco’s modified Eagle’s medium (DMEM; PAN-Biotech, Aidenbach, Germany), containing pyruvate for SK-Mel-29 and pyruvate-free for SaOS-2. Media were supplemented with 10% fetal calf serum (FCS; PAN-Biotech, Aidenbach, Germany), 0.1 mM non-essential amino acids, 50 U/mL penicillin, and 50 µg/mL streptomycin. All cell lines were authenticated and routinely tested for mycoplasma contamination using the Venor^®^ GeM Classic kit (Minerva Biolabs, Berlin, Germany). Adenoviral vectors encoding TAp73α, DNp73β (encoding ΔEx2/3p73β), or GFP have been reported previously [5]. Infections of SK-Mel-29 were performed at a multiplicity of infection selected to achieve 100% transduction of the target cells. SaOS-2 cells were transfected with pcDNA3.1-TAp73α or pcDNA3.1-DNp73β as previously described [5] using TurboFect (Thermo Scientific, Waltham, MA, USA) according to the manufacturer’s instructions.

### 2.2. Immunoblotting

Protein lysates were prepared and subjected to immunoblotting as previously described [8]. Membranes were incubated with primary antibodies against p73 (ER-15, 1:1000; Thermo Scientific, Waltham, MA, USA, RRID:AB_1077442) and β-actin (1:5000; Sigma Aldrich, St. Louis, MO, USA, RRID:AB_476692).

### 2.3. Co-Immunoprecipitation (Co-IP) and Mass Spectrometry (MS)

For both Co-IP and Co-IP–MS, 0.5 mg of precleared cell lysate (cleared from unspecific binding by incubation with Protein G Sepharose beads (Cytiva, Marlborough, MA, USA) was incubated overnight at 4 °C with anti-p73 antibody (ER-15; Thermo Scientific, Waltham, MA, USA, RRID:AB_1077442). Protein-protein complexes were captured with preblocked Protein G Sepharose beads for 1.5 h at 4 °C, washed extensively and eluted by heating at 95 °C for 10 min. For Co-IP, eluates together with 0.05 mg input lysate were separated by SDS–PAGE and immunoblotted with antibodies against p73 and β-actin. For CoIP-MS, full LC-MS/MS settings have been published previously [5].

### 2.4. Cleavage Under Targets and Release Using Nuclease (CUT&RUN)

CUT&RUN assays were conducted on SK-Mel-29 melanoma cells transduced with Ad.TAp73α, Ad.DNp73β or the Ad.GFP control, following the manufacturer’s protocol (CUT&RUN Assay Kit, Cell Signaling, Danvers, MA, USA). Immunoprecipitation was performed with p73 antibody (Thermo Scientific, Waltham, MA, USA, RRID:AB_1077442), and libraries were prepared with the DNA Library Prep Kit for Illumina Systems (Cell Signaling, Danvers, MA, USA) according to the instructions. Library quality was assessed by fragment-length profiling using the Agilent High Sensitivity DNA Kit (Agilent, Santa Clara, CA, USA), and DNA concentration was determined with the Qubit dsDNA High Sensitivity Assay Kit (Thermo Scientific, Waltham, MA, USA). Libraries were multiplexed and sequenced at a depth of 10 million reads per sample in paired-end mode (2 × 150 bp) at a loading concentration of 750 pM on a NextSeq 1000/2000 system using a P1 flow cell. Raw sequencing reads (FASTQ) were generated with DRAGEN BCL Convert (https://support.illumina.com/downloads/dragen-workflow-bclconvert-installer.html, accessed on 21 July 2025) and quality-checked using FastQC (https://www.bioinformatics.babraham.ac.uk/projects/fastqc/, accessed on 21 July 2025). Preprocessing was carried out with Trim Galore version 0.6.10 (https://www.bioinformatics.babraham.ac.uk/projects/trim_galore/, accessed on 21 July 2025); low-quality reads (Phred score < 20) and short reads (<20 bp) were removed, and 3′ adapter-like sequences (Illumina, San Diego, CA, USA) were trimmed. All CUT&RUN data were processed through the nf-core (doi: 10.1038/s41587-020-0439-x) CUT&RUN pipeline version 3.2.2; (doi: 10.5281/zenodo.5653535) to ensure reproducibility and standardization.

### 2.5. Microarrays and Gene Set Enrichment Analysis (GSEA)

Total RNA was isolated with the NucleoSpin RNA kit (Macherey-Nagel, Düren, Germany) and converted to cDNA using the First Strand cDNA Synthesis Kit (Thermo Scientific, Waltham, MA, USA). For expression profiling, RNA from triplicate SK-Mel-29 samples transduced to express TAp73α, DNp73β, or GFP was hybridized to Clariom™ D arrays (Thermo Scientific, Waltham, MA, USA). Probe intensities were background-adjusted, processed, and normalized in Transcriptome Analysis Console (TAC 4.0) with the SST-RMA procedure. Differentially expressed genes (DEGs) relative to the controls were identified [5], applying *p* < 0.05 and Fold Change (FC) ≥ 1.5. Gene set enrichment analysis (GSEA) was carried out with GSEA v4.3.3 against curated MSigDB collections (https://software.broadinstitute.org/gsea/msigdb/, accessed on 21 July 2025), using the Hallmark database (h.all.v2024.1.Hs.symbols). The following parameters were adjusted from default: number of permutations = 1000; permutation type = gene set; chip platform = Human_Clariom-D.r1_MSigDB.v2025.Hs_REMAPPED-PATCH; enrichment statistic = weighted; metric for ranking genes = log2_ratio_of_classes. Enriched hallmark terms were visualized against the negative log10 of their individual FDR values (<0.05).

### 2.6. Determination of Isoform-Specific Coregulators of p73 Binding

The analysis was anchored on the p73 binding motif from the JASPAR transcription factor database (MA0861.1; https://jaspar.elixir.no/, accessed on 21 July 2025; 2022 release) [9], which was optimized in vitro to represent the DNA-binding characteristics common to all p73 isoforms [10]. All JASPAR motifs were mapped to the human reference genome GRCh38 (Ensembl v112). To this end, we implemented a C++ program that processes each column of a JASPAR-provided position-specific scoring matrix (PSSM) by calculating the relative nucleotide frequencies. To score a genomic position, the program sums the −log_2_ values of the nucleotides specified by the PSSM columns and subtracts the baseline score expected for a non-informative column (equal nucleotide frequencies of ¼, corresponding to a score of 2 per position). All sites with a total score ≥ 0 were retained as candidate binding sites for the respective TF. Using a sliding-window approach, genome-wide match files were generated for each JASPAR motif.

From these genome-wide matches, we constructed a comprehensive summary table. For each predicted p73 site, all other JASPAR TF binding sites within ±100 bp and a respective score > 0 were cataloged, including their frequency, the maximum binding score, and the distance of the strongest match relative to the p73 site. Promoter regions, defined from the Ensembl v112 GTF annotation as the 500 bp upstream of any transcript start site, were flagged in a dedicated column to support optional filtering.

Each 18 bp JASPAR-derived site was further annotated with the minimum CUT&RUN coverage, defined as the lowest number of overlapping reads at that position. This metric was chosen to help distinguish closely spaced p73 sites: when one site coincided with a CUT&RUN peak and an adjacent site did not, read coverage initiating within the flanking site indicated endonuclease accessibility, suggesting that the site was unbound. CUT&RUN coverage values were extracted from bigWig files generated by the nf-core pipeline and processed with BEDTools [11].

### 2.7. Downstream Analyses and Visualizations

All downstream analyses were performed in R version 4.3.3 (https://www.r-project.org) using the summary table of predicted p73 binding sites and their associated JASPAR motif frequencies within ±100 bp. Transcription factor enrichment was quantified as the log ratio of motif frequencies before and after applying a given filter to the p73 binding sites. Visualizations were generated using ggplot2 version 3.5.2 for scatter plots (10.32614/CRAN.package.ggplot2), pheatmap version 1.0.12 for heatmaps (10.32614/CRAN.package.pheatmap), and corrplot version 0.94 for correlation matrices (10.32614/CRAN.package.corrplot) [12,13,14]. The UpSet plot were generated using the online platform UpSetR (Gehlenborg Lab; https://gehlenborglab.shinyapps.io/upsetr/, accessed on 21 July 2025) as described previously [15].

### 2.8. Expression, Survival, and Principal Component Analysis in Patient Data

Gene expression and survival analyses were performed using GEPIA2 (http://gepia2.cancer-pku.cn, accessed on 21 June 2025), which integrates TCGA and GTEx datasets [16]. Skin Cutaneous Melanoma (SKCM) was selected as the cancer type. For expression analysis, melanoma samples were compared with normal skin (including GTEx data) using the Box Plot tool (cutoffs: log_2_FC = 1, *p* = 0.01, jitter size = 0.4). For survival analysis, overall survival was assessed with default parameters (group cutoff: median; 95% confidence interval), and genes significantly associated with outcome (*p* < 0.05) were considered clinically relevant. In addition, the “Most Differential Survival Genes” tool was used to extract the top 500 genes with the strongest positive impact on patient survival (survival dependency). Survival maps were generated from overall survival data using median cutoffs for selected gene signatures, with hazard ratios (HR) estimated by Mantel–Cox test. Finally, principal component analysis (PCA) was conducted on TCGA SKCM and GTEx sun-exposed and non–sun-exposed skin samples, with results displayed as two-dimensional plots.

## 3. Results

### 3.1. Genome-Wide Detection of DNA Binding Profiles of TAp73 and DNp73 via Integrative CUT&RUN-JASPAR-Based Approach

**Figure 1:**
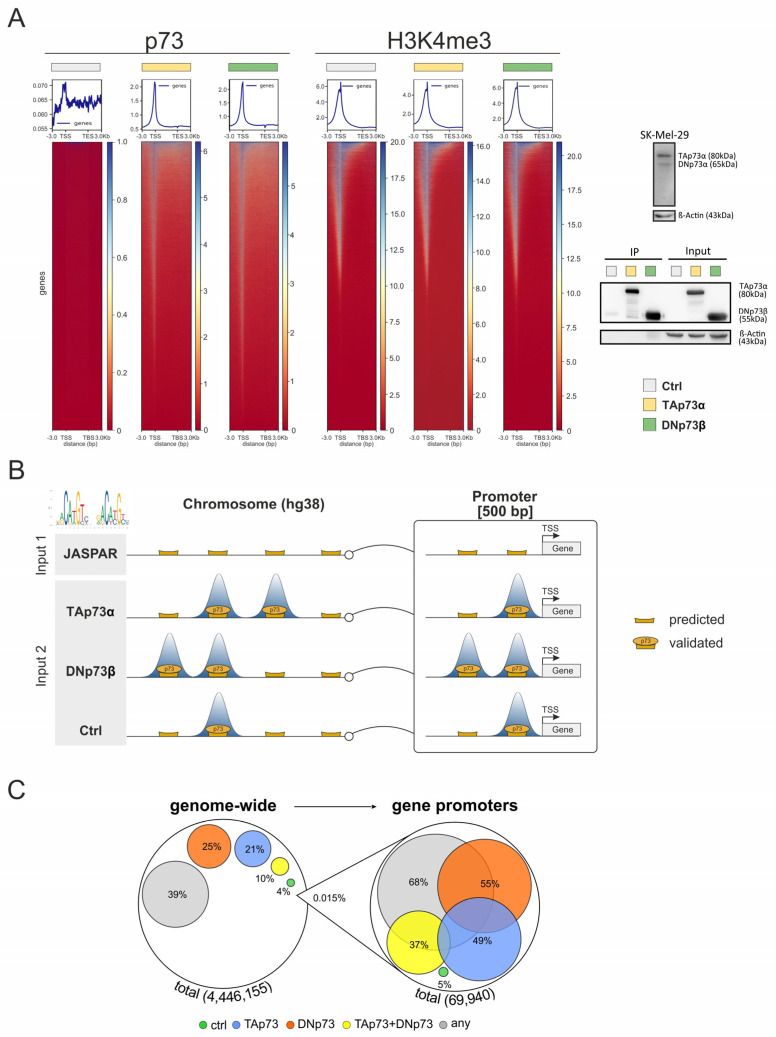
Integrative CUT&RUN and Motif Analysis of TAp73α and DNp73β Binding Sites. (**A**) CUT&RUN read density maps of p73 (left) and H3K4me3 (right) peaks centered on genomic regions spanning annotated transcription start (TSS) and end sites (TES) with ±3 kbp flanks, in SK-Mel-29 cells expressing TAp73α, DNp73β, or control (ctrl). A Western blot indicating endogenous p73 isoform levels in SK-Mel-29 cells and p73 immunoprecipitation (IP) with corresponding input samples for control, TAp73α-, and DNp73β after overexpression is shown in panel (**A**) (right site). β-Actin was used as loading control for normalization. (**B**) Workflow integrating JASPAR motif predictions (Input 1) with CUT&RUN sequencing for p73 isoforms (Input 2) to define high-confidence p73 binding sites. Binding sites were analyzed genome-wide and specifically within promoter regions (500 bp upstream of the transcription start site, TSS). (**C**) Quantity chart of p73 binding sites predicted by JASPAR and confirmation by CUT&RUN in SK-Mel-29 expressing TAp73 or DNp73. Colored circles show the percentage of each analyzed condition compared to the total amount of identified p73 site. Yellow circles refer to the relative amount of p73 sites detected in both TAp73 and DNp73 sets (blue for TAp73, orange for DNp73), whereas gray circles indicate confirmed binding in at least one of the three groups. Overlapping areas do not represent the commonalities between groups.

To investigate the genome-wide binding behavior of specific p73 isoforms, we performed CUT&RUN in SK-Mel-29 following overexpression of either TAp73α or DNp73β, and compared the profiles to the controls. In a first step, p73 binding was mapped across genomic regions containing annotated genes plus flanking 3 kbp including both transcriptional start site (TSS) and transcriptional end site (TES) of each gene (Figure 1A, left). In contrast to the strong, sharply localized peaks observed upon overexpression of the isoforms, p73 binding was, due to the lower endogenous p73 levels, barely detectable in control cells. These levels were insufficient for reliable antibody pulldown, underscoring the need for overexpression for accurate detection of binding sites (Figure 1A, right, Supplementary Tables S1 and S2). Importantly, CUT&RUN profiling of the active histone mark H3K4me3, which was used as a positive control, showed comparable signal intensities and global distribution patterns for all three conditions (TA, DN and control; Figure 1A, center). This indicates similar TF accessibility to endogenous DNA binding sites and confirms that isoform-specific chromatin binding occurs within a stable epigenetic landscape, supporting the physiological relevance of the observed interactions. These results corroborate the robustness of our approach and provide a consistent framework for downstream analyses of isoform-specific cofactor interactions and transcriptional regulation.

Next, to define the exact location of highly reliable p73 binding sites, we searched for matches to the JASPAR-derived p73 consensus motif [10] (Figure 1B). Using the position-specific scoring matrix (PSSM), we scanned the entire genome (~3 billion base pairs) and calculated a log-likelihood ratio score for every genomic location. To score each position, the observed nucleotide frequency as denoted in the PSSM was related to a uniform background distribution (25% per nucleotide), and the resulting log-likelihood values were summed across the motif. A score of zero or less corresponds to a randomly expected match, while positive scores indicate enrichment relative to the background. This analysis identified approximately four million putative p73 binding sites, which were then compared with the obtained CUT&RUN data to confirm and quantify DNA binding with full coverage of the binding regions (Figure 1C).

Overall, 39% of the binding sites derived from JASPAR were found to be completely covered by at least one read of the CUT&RUN sequencing data for at least one isoform. For the scope of this manuscript, we interpret the overlapping locations as a tentative confirmation of these JASPAR-predicted binding sites by CUT&RUN. This proportion is significantly higher than the 11% of the genome covered by a CUT&RUN read, demonstrating that (i) motif strength alone cannot reliably predict actual binding, and (ii) the p73 JASPAR motif and its genomic mapping are biologically relevant. Under our experimental conditions, 21% of these sites were specifically bound by TAp73α, 25% by DNp73β, and 10% by both isoforms. Notably, when focusing on promoter-proximal regions defined as 500 bp upstream of TSS, 68% of all predicted sites were located within a mapped CUT&RUN read, with 49% bound by TAp73α, 55% by DNp73β, and 37% by both isoforms. Consistent with the enrichment profiles shown in Figure 1A, these results show the preferential recruitment of p73 to regulatory elements near TSSs.

### 3.2. Positive and Negative TF Motif Enrichment Defines Potential p73 Regulatory Factors

**Figure 2:**
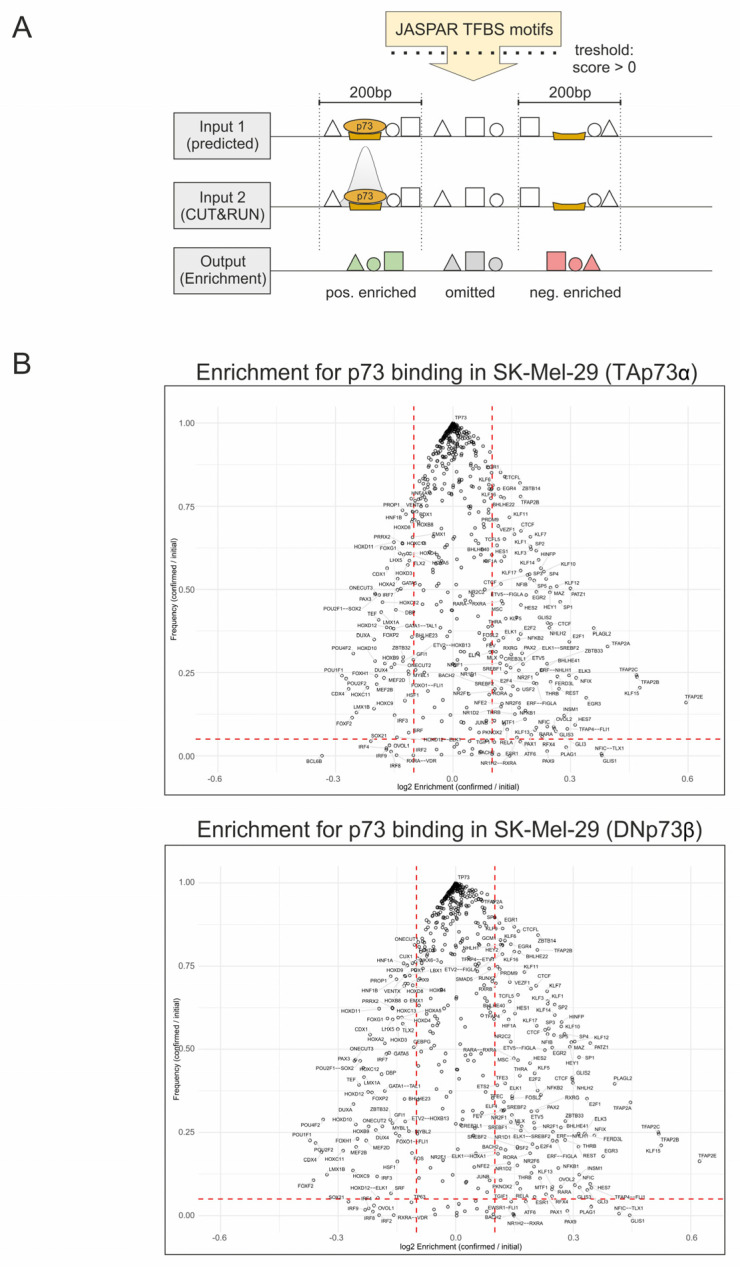
Genome-wide Enrichment Analysis of Transcription Factor Co-Localization at p73 Binding Sites. (**A**) Overview of the analysis strategy: 608 JASPAR-derived transcription factor binding sites (TFBS) were mapped to the human genome, generating two inputs. Input 1 contains all predicted TFBS, including the p73 motif. Input 2 contains all predicted TFBS, as well as the CUT&RUN-confirmed p73 sites. Subsequently, the enrichment was calculated as log2 ratio of the indicated inputs within a ±100 bp window around the p73 sites and plotted against the motif frequency. (**B**) Swarm plot of TFBS enrichment at confirmed p73 binding sites for TAp73α (top) and DNp73β (bottom). Dashed red lines indicate the significance threshold values: log_2_ Enrichment ENR > 0.1, Frequency (F) > 0.05. ENR enrichment; F Frequency.

Because of established interactions of p73 with transcription factor Sp1 and its DNA binding sites [17] and our own work on an isoform-specific interaction with REST [5], we assumed that the function of p73 isoforms is massively determined by transcriptomic coregulators that bind to and interact with TA or DN in close proximity to the detected p73 TF motifs of target gene promoters. To identify potential coregulatory transcription factors (coTFs), we performed a genome-wide enrichment analysis of all 608 JASPAR-annotated human TF motifs located within ±100 bp of the four million predicted p73 binding sites (Figure 2A). Enrichment was calculated as the log_2_ ratio of relative TF motif frequency at experimentally validated CUT&RUN p73 sites versus all predicted p73 sites and plotted against motif frequency. As shown in Figure 2B, this analysis yielded two essential results: (i) positive enrichment (log_2_ > 0), where TF motifs occurred more frequently than predicted near confirmed p73 sites, suggesting that these factors may act as potential coTFs or cofactors that facilitate p73 binding. (ii) negative enrichment (log_2_ < 0), where TF motifs near confirmed sites occurred less frequently than expected, implying depletion or competition, for instance their presence could reduce the likelihood of p73 occupancy. Consequently, the p73 motif itself shows no enrichment (log_2_ = 0) and the highest frequency. Moreover, the distribution patterns were similar for both conditions, with no significant differences between TA and DN. In the group of most positively enriched candidates, we found motifs for coTFs TFAP2A/B/C/E, KLF15, EGR3, FERD3L, PLAGL2, GLIS1, NFIX, PATZ1, E2F1, PLAG1, HES7, ELK3, SP1, REST and INSM1, whereas the TFBSs of POU1F1, POU2F2, CDX4, FOXF2, DUXA, HOXC9, HOXA and HOXD showed strong negative enrichment. All negatively enriched genes are controlling embryonic development, and so does p73 [2,18,19,20].

### 3.3. Integration of Binding and Expression Data Identifies Isoform-Specific CoTF Networks

**Figure 3:**
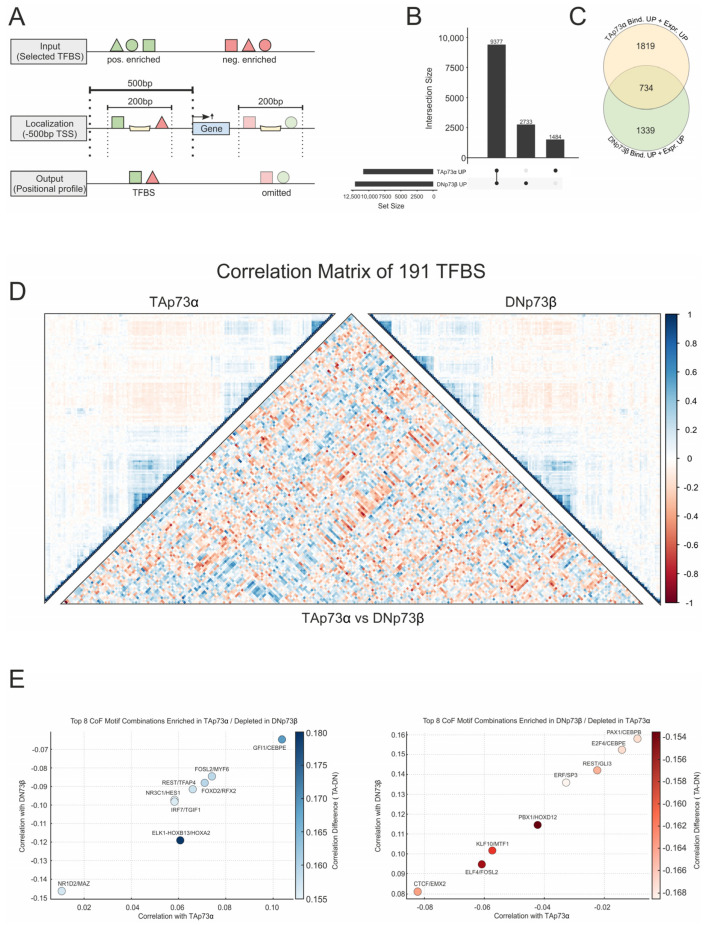
Integration of binding and expression data reveals isoform-specific coregulator motif networks of p73. (**A**) Illustration of the co-binding analysis strategy: TFBS (from JASPAR) located within ±100 bp of confirmed p73 binding sites in the promoter region were assessed for their co-localization with p73 isoforms based on CUT&RUN data. (**B**) UpSet plot showing the number of genes with confirmed promoter-proximal binding of TAp73α and DNp73β. (**C**) Comparison of isoform-specifically bound genes with increased expression (FC ≥ 1.5): 1819 for TAp73α, 1339 for DNp73β, and 734 common between both isoforms. (**D**) Correlation matrix of 191 TFBS enriched at p73-bound promoters of DEGs identified in (**C**). Left and right panels show isoform-specific coregulator associations for TAp73α and DNp73β, respectively, while the central matrix highlights differences in motif co-occurrence between the two isoforms. Blue indicates TAp73α and red DNp73β enrichment. log_2_ENR > 0.1, F > 0.05 (**E**) Scatterplots of the top eight coTF motif pairs for TAp73α and DNp73β that show the strongest differences in correlation selected from (**D**) using two complementary ranking strategies: (left) TAp73α enriched vs. DNp73β depleted, and (right) DNp73β enriched vs. TAp73α depleted. Each point corresponds to a cofactor combination, with color intensity indicating the absolute correlation difference, highlighting the most isoform-specific associations.

To further investigate how coTF enrichment relates to functional differences between TA and DN isoforms, we integrated p73-dependent gene expression analyses from melanoma cells following TAp73α or DNp73β overexpression (DEGs; FC > 1.5; Figure 3A) into the CUT&RUN dataset that contained confirmed p73 binding sites in their promoters (Figure 1C). Both isoforms showed a strong enrichment for promoter-associated binding, with 9377 genes bound by both, and 1484 and 2733 specifically targeted by TAp73α or DNp73β, respectively (Figure 3B). Strikingly, by integrating expression and binding data, we identified 1819 TAp73α-specific and 1339 DNp73β-specific DEGs, while only 734 genes are both bound and upregulated by TA and DN (Figure 3C). This demonstrates that combining transcriptomic and binding data refines the pool of functional p73 target genes and reveals clear isoform-specific regulatory programs.

Building on these findings and analogously to the earlier enrichment analysis, we then searched for promoter-proximal coTF motifs among upregulated genes. We calculated the log ratio by comparing TFBS frequencies within ±100 bp of p73-bound sites in DEGs with their frequency in all promoters. This yielded 191 promoter-enriched TF motifs associated with TAp73α- and DNp73β-regulated genes.

As shown in Figure 3D, the overall landscape of coregulatory motifs between TAp73α (left matrix) and DNp73β (right matrix) appears largely similar. However, a direct comparison of the correlation values (central matrix) revealed distinct isoform-specific motif associations. To clarify the differences in coTF motifs between TA and DN, we ranked the motif combinations in two ways: (i) those that are enriched under TAp73α but depleted for DNp73β, and (ii) those enriched under DNp73β but depleted for TAp73α. The top eight isoform-specific coregulation motifs are shown in Figure 3E. Prominent examples are ELK1::HOXB13/HOXA2, GFI1/CEBPE, and FOXD2/RFX2 (Figure 3E, left), which may represent TAp73α-specific coregulator networks that could be competitively inhibited or excluded in the presence of DNp73β, possibly through cofactor sequestration, chromatin remodeling, or restricted site accessibility. Importantly, several of the identified motifs correspond to JASPAR motifs already associated with p73-dependent enrichment in Figure 2B.

We recently reported that REST interacts with TAp73 via HDAC2 [5]. Our current findings, indicating the co-occurrence of TFAP4/REST motifs at TAp73 sites, while DN shows enrichment of REST/GLI3, suggest differential recruitment strategies that may either promote or inhibit this p73 protein–protein interaction. Conversely, motif pairs such as ERF/SP3, PAX1/CEBPB, and E2F4/CEBPE (Figure 3E, right) were preferentially enriched under DNp73β but suppressed under TAp73α, again reflecting isoform-specific coregulator engagement. Together, this underscores that although TAp73α and DNp73β bind largely overlapping genomic regions, they can recruit distinct sets of coregulators. This highlights the complexity of isoform-specific transcriptional regulation and a context-dependent assembly of p73-centered regulatory complexes.

### 3.4. Functional Dissection of TA/DN-Specific Coregulator Recruitment in Cancer-Hallmark Pathways

**Figure 4:**
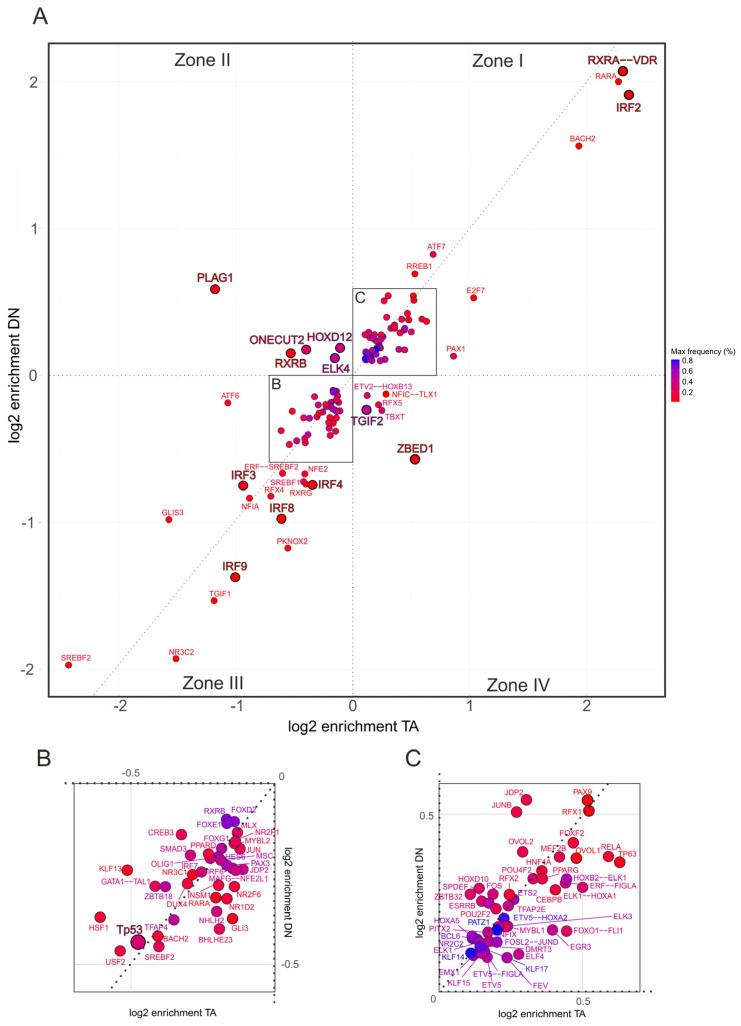
Promoter-specific motif enrichment within the EMT hallmark gene set. (**A**) Two-dimensional scatterplot showing log_2_ enrichment of TF binding site motifs at CUT&RUN-confirmed TAp73α (x-axis) and DNp73β (y-axis) peaks located in the 500 bp promoter regions of hallmark genes. Motifs are classified into four zones: Zone I—shared motifs with isoform bias (above diagonal: DN-preferred; below diagonal: TA-preferred); Zone II—DNp73β-specific; Zone III—depleted in both isoforms; Zone IV—TAp73α-specific. (**B**,**C**) Insets show magnified regions from panel (**A**), highlighting motifs depleted in both isoforms (**B**) or co-enriched by both isoforms (**C**), facilitating interpretation of less prominent but potentially relevant coregulators. Each dot represents one motif; color intensity reflects motif frequency near p73 peaks (±100 bp), providing a measure of both abundance and enrichment.

**Figure 5:**
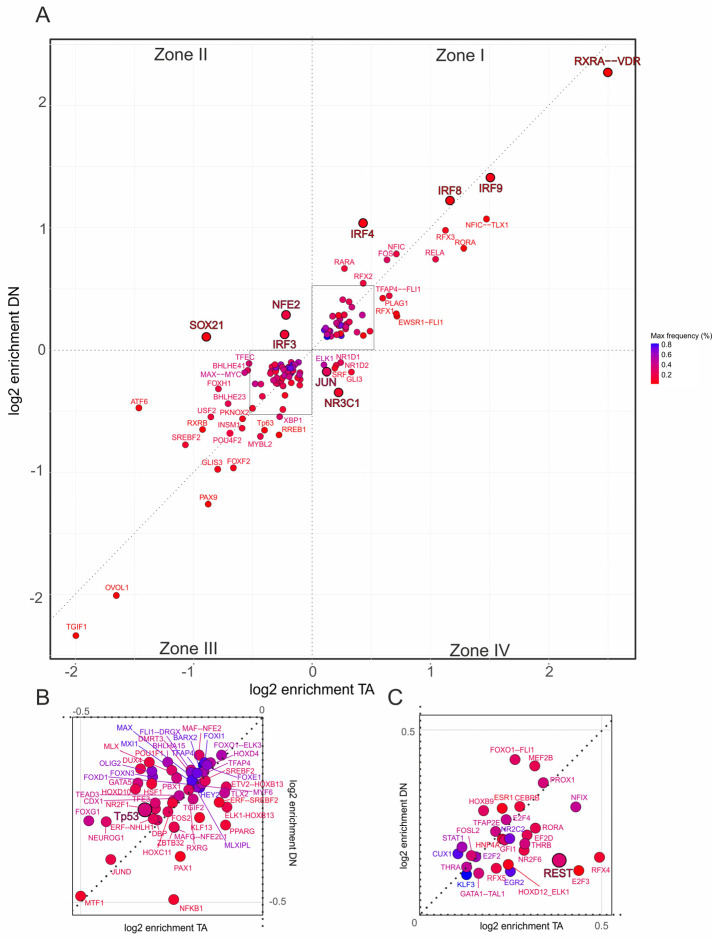
Promoter-specific motif enrichment within the inflammatory response hallmark gene set. (**A**) Two-dimensional scatterplot showing log_2_ enrichment of TF binding site motifs at CUT&RUN-confirmed TAp73α (x-axis) and DNp73β (y-axis) peaks located in the 500 bp promoter regions of hallmark genes. Motifs are classified into four zones: Zone I—shared motifs with isoform bias (above diagonal: DN-preferred; below diagonal: TA-preferred); Zone II—DNp73β-specific; Zone III—depleted in both isoforms; Zone IV—TAp73α-specific. (**B**,**C**) Insets show magnified regions from panel (**A**), highlighting motifs depleted in both isoforms (**B**) or co-enriched by both isoforms (**C**), facilitating interpretation of less prominent but potentially relevant cofactors. Each dot represents one motif; color intensity reflects motif frequency near p73 peaks (±100 bp), providing a measure of both abundance and enrichment.

Having established that TAp73α and DNp73β engage distinct coregulator motif networks on a genome-wide scale, we examined whether these differences affect cancer driver pathways. We focused on hallmark gene sets associated with cancer-related processes, including EMT and inflammatory signaling. To facilitate interpretation of the resulting two-dimensional scatterplots in Figure 4 and Figure 5, we divided the motifs into four zones according to their relative log_2_ enrichment under TAp73α and DNp73β. Zone I represents motifs positively enriched at p73-bound promoters, whereas Zone III captures negatively enriched motifs. In both zones, positioning along the diagonal indicates stronger enrichment for either DNp73β (above diagonal) or TAp73α (below diagonal). In contrast, Zones II and IV indicate motifs enriched under one isoform but depleted under the other, allowing isoform-specific coregulator associations to be precisely determined.

Importantly, the analysis revealed different p73 coregulator patterns depending on the gene set. For EMT (Figure 4A), DNp73β dominates co-enrichment with motifs for PLAG1, RXRB, ONECUT2, ELK4, and HOXD12, with HOXD12 being also identified in the genome-wide analysis (Figure 3E, right). In contrast, motifs such as NFE2, IRF3, and SOX21 are specific to DNp73β in the inflammatory-response trait (Figure 5A). Similarly, TAp73α exhibits distinct motif sets for each hallmark, including ZBED1 and TGIF2 for EMT or JUN and NR3C1 in case of inflammatory response.

Several factors display strong positive or negative enrichment exclusively in Zone I or III and are specific to a particular hallmark. Among these, the RXRA::VDR motif shows the highest enrichment with a preference for TAp73α (log_2_ > 2) and is the only motif that occurs for both traits. Furthermore, IRF2 is enriched for TAp73α in EMT but is completely absent in the immune-relevant gene set. Other members of the IRF-family such as IRF3, IRF4, IRF8, and IRF9 switch from negative enrichment in the EMT to positive enrichment in the inflammatory response cluster, with a tendency toward DNp73β. Interestingly, among all identified p73 interactors, REST was the only factor with detectable enrichment that occurred selectively in inflammatory response genes (Figure 5C), suggesting a possible role of the TAp73-REST interaction in the neuro-immune crosstalk of melanoma cells.

We also observed distinct regulatory patterns for the p73 family members p53 and p63. p53 motifs are overall depleted (Figure 4B and Figure 5B), which is consistent with competitive binding to p73 that can lead to opposing functional outcomes. p63 motifs are positively enriched in EMT with a clear preference for TAp73α (Figure 4C), but shift to negative enrichment in the inflammatory response trait (Figure 5A). Given the close structural and functional relationship between p63 and p73, this suggests that truncated p63 isoforms could impair the tumor suppressor role of TAp73α and alter cancer-relevant gene regulatory pathways, leading either to gain-of-function effects in EMT or loss-of-function effects in inflammatory responses.

In sum, our motif analysis shows that TAp73α and DNp73β share an extensive repertoire of coTFs, but their preferences diverge depending on the functional context. This isoform-specific coTF usage becomes particularly evident when compared with the genome-wide analysis (Figure 2B), where many of these TFs are similarly distributed between the isoforms.

### 3.5. Coregulator Architecture Governs TA and DN EMT Programs

**Figure 6:**
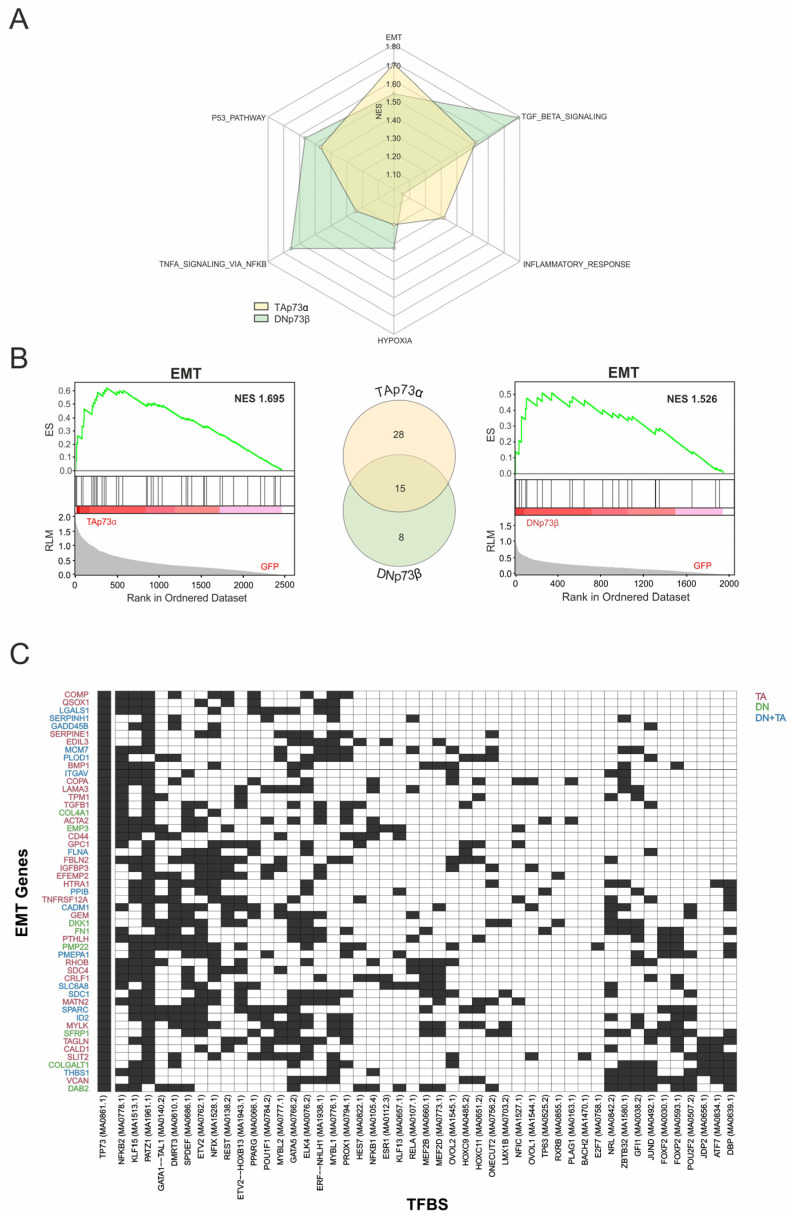
Isoform-Specific p73 Targets Reveal EMT-Associated Coregulators. (**A**) Radar plot comparing Hallmark gene sets enriched in the subset of genes regulated by both TAp73α and DNp73β. Axes correspond to selected Hallmark terms, while color intensity and distance from the center indicate the normalized enrichment score (NES). (**B**) GSEA enrichment plots depict EMT Hallmark gene set upregulation under TAp73α (left) and DNp73β (right) overexpression (NES = 1.69 and 1.53, respectively). The central Venn diagram highlights the overlap between isoform-specific EMT genes, with 28 unique to TAp73α, 8 unique to DNp73β, and 15 shared. (**C**) Heatmap of TFBS enriched near confirmed p73 binding sites in significantly upregulated EMT-hallmark genes. Rows represent genes and columns show the 25 most-enriched motifs for each isoform-specific EMT set (TAp73α, DNp73β, or shared), across 51 EMT genes. Black cells indicate motif presence near a p73 site, white cells indicate absence. Gene labels are color-coded by isoform association: red (TAp73α), green (DNp73β), and blue (shared).

To gain deeper insight into the contribution of p73-coTF interactions to cancer phenotypes, we concentrated on upregulated genes with confirmed p73 promoter binding (Figure 3C) and subjected them to gene set enrichment analysis (GSEA). Normalized enrichment scores (NES) revealed isoform-triggered signatures, indicating that TAp73α-regulated genes are more strongly associated with EMT and inflammatory response, whereas DNp73β showed higher enrichment for TNFα- and TGFβ-signaling pathways (Figure 6A). Regarding EMT-related genes, GSEA confirmed significant enrichment for both isoforms (TAp73α: NES = 1.69; DNp73β: NES = 1.53), whereas subsequent comparative analysis identified 28 EMT genes that were exclusively upregulated and bound by TAp73α, 8 specific to DNp73β, and 15 common to both (Figure 6B). Due to this significant overlap, we next performed a coTF motif enrichment analysis for the entire set of 51 genes. Among the most significantly enriched motifs near p73 sites are PATZ1 (47 genes), KLF15 (31), NFKB2 (27), ELK4 (25), and ETV2 (25). Conversely, several coregulators were selectively associated with only a few target genes, including E2F7 and BACH2 (1 each), RXRB and PLAG1 (2 each), and TP63 (3). Distinct regulatory architectures were also evident, with some genes acting as hubs with numerous enriched coregulators in close proximity to p73 binding sites (e.g., SFRP1, DAB2 with 20 motifs each, MYLK with 20, VCAN and SPARC with 18 each), while others displayed focal signatures with only a few enriched motifs (e.g., GADD45B [6], COL4A1 [7], SERPINH1 [7], TPM1 [8], FLNA [9]) (Figure 6C). However, we also discovered coTFs with negative enrichment in the EMT gene set such as MEF2B and POU2F2 (Figure 2B). Their presence among EMT-associated genes corroborates that both positively and negatively enriched coregulators contribute to the isoform-driven regulatory network. These data suggest that EMT genes are controlled through a multi-layered regulatory architecture in which common hubs ensure core activation of this cancer hallmark, while coTFs may fine-tune the functional outcomes of TAp73α and DNp73β.

### 3.6. EMT Coregulator Signatures Predict Patient Survival in Skin Cancer

**Figure 7:**
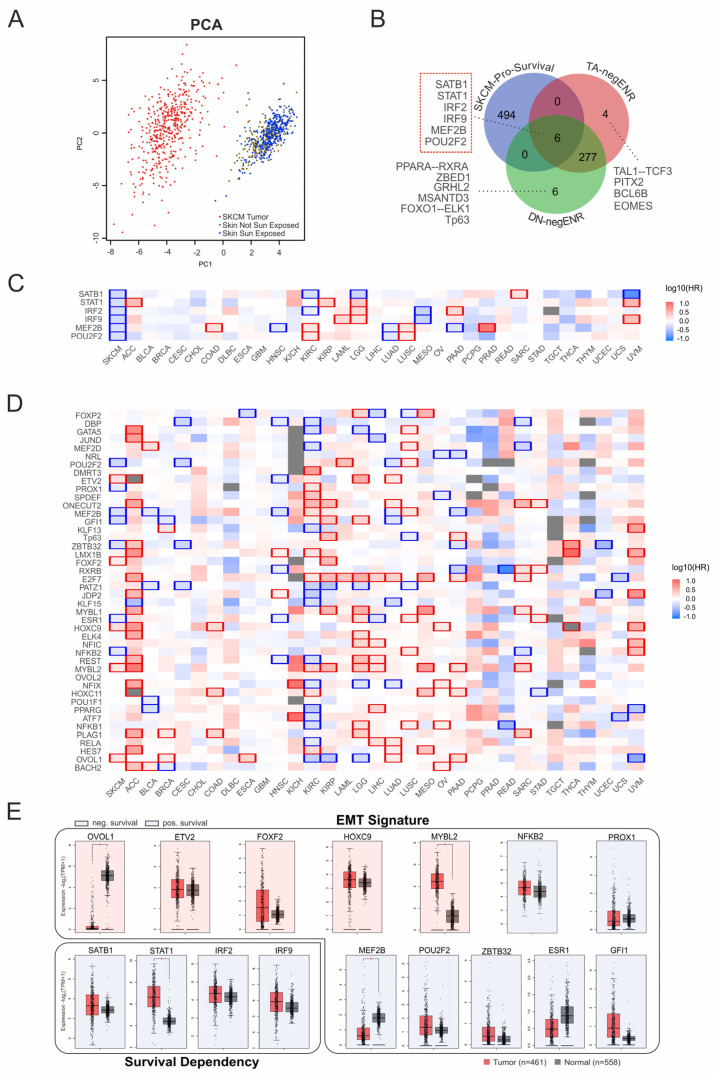
Survival dependency of EMT-associated coTFs. (**A**) Principal component analysis of p73-associated EMT cofactors separates melanoma samples (red) from healthy skin, including sun-exposed (blue) and non–sun-exposed (brown) subgroups. PC1 (x-axis) and PC2 (y-axis) represent the first and second principal components derived from log_2_(TPM + 1) expression values. (**B**) Venn diagram illustrating the overlap between genes with the strongest positive impact on survival in SKCM and coTF motifs negatively enriched for TAp73α and DNp73β. Relevant factors (motifs) are indicated. (**C**) Heatmap depicting the survival dependency of TAp73α- and DNp73β–common negatively enriched coregulators identified in (**B**) across melanoma (SKCM) and other cancer types. (**D**) Heatmap showing the survival dependency of EMT-associated factors (from Figure 6C) across cancer types (x-axis, TCGA abbreviations). Colors indicate hazard ratios (HR), with blue representing high expression linked to improved survival and red indicating high expression associated with poor survival. (**E**) Analysis of expression patterns of survival (**C**) and EMT-related genes (**D**) in tumor samples (red boxes) vs. healthy tissue (grey boxes). Red/blue shading indicates poor/good prognosis associated with the genes. * *p* < 0.05.

To evaluate the clinical relevance of the identified coregulators, we next tested the motif signatures of EMT genes in patient datasets. Principal component analysis using the coTF motif-defined signature successfully separated skin cancer samples from healthy tissue, confirming its quality as a classifying marker (Figure 7A). Since several coTFs also showed negative enrichment in the global motif analysis (Figure 2B), we assessed their potential impact on patient survival. For this, we compared the negatively enriched TFBS specific to TAp73α and DNp73β with a list of genes connected to favorable survival outcomes in melanoma (Figure 7B). This analysis yielded six candidates, all of which showed significant positive effects on survival in skin cancer. Importantly, the same genes exhibited variable associations in other cancer types (Figure 7C), underscoring the context-dependent nature of coregulator functions.

From the EMT gene set (Figure 6C), seven candidates are associated with favorable outcomes (*MEF2B*, *POU2F2*, *NFKB2*, *ESR1*, *ZBTB32*, *GFI1*, *PROX1*), while five correlate with poor survival (*OVOL1*, *MYBL2*, *HOXC9*, *FOXF2*, *ETV2*). This suggests that certain factors could serve as therapeutic targets to promote favorable outcomes by attenuating EMT, whereas targeting other factors could worsen prognosis. Notably, this pattern was highly specific to skin cancer, as several factors showed opposite associations in other cancer types (Figure 7D).

In order to prioritize candidates for regulators, we examined their expression patterns in patient samples versus healthy tissue, including the survival genes *SATB1*, *STAT1*, *IRF2*, and *IRF9*. Strikingly, significant expression changes were observed only for a subset of these genes, with *STAT1* upregulated and *MEF2B* downregulated in the group with favorable survival, while *MYBL2* was increased and *OVOL1* reduced in the unfavorable group (Figure 7E). This indicates that expression changes alone cannot fully explain survival dependencies and may even obscure functionally relevant factors. Our data rather suggest that the regulatory context and network integration determines whether a particular factor contributes to tumor progression or suppression.

### 3.7. Integrative Analysis of p73 Cofactors Identifies PATZ1 as DNp73 Interactor

**Figure 8:**
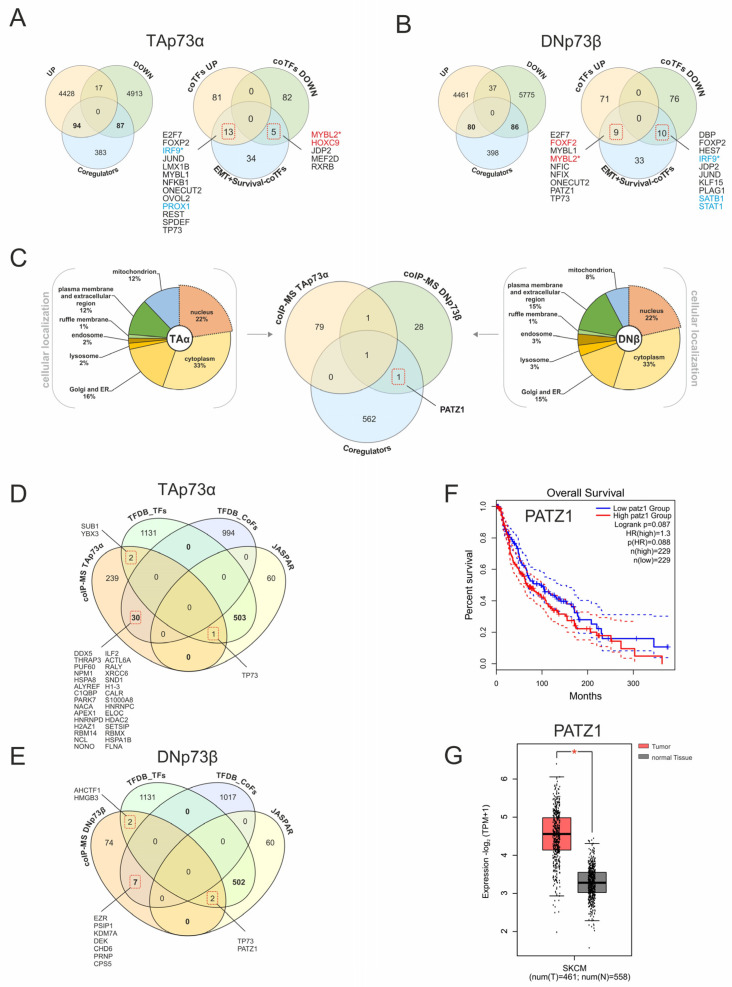
Isoform-specific transcriptomic and proteomic landscapes of p73 coregulators reveal PATZ1 as a DNp73β interactor. (**A**,**B**) Comparative analysis of expression data for TAp73α (**A**) and DNp73β (**B**) with CUT&RUN-confirmed coTFs, EMT signature genes, and survival-dependency genes. A total of 564 unique factors were compiled after removal of duplicates, as several JASPAR-derived motifs correspond to the same transcription factor. Genes associated with negative survival are highlighted in red, while prosurvival genes are shown in blue. Asterisks highlight the genes oppositely regulated by both p73 isoforms. (**C**) Cellular localization and overlap of nuclear-localized proteins identified by CoIP-MS with TAp73α and DNp73β. The Venn diagram illustrates the overlap between CoIP-MS-detected candidates and predicted coregulators. (**D**,**E**) Comparison of TFDB-annotated transcription factors and cofactors with JASPAR-derived factors and CoIP-MS–identified interactors. (**F**) Kaplan–Meier survival plot of *PATZ1* expression in melanoma patient samples. (**G**) *PATZ1* expression levels in TCGA skin cancer (SKCM) dataset. Solid lines represent the survival curves, while dashed lines indicate the 95% confidence intervals for each group. * *p* < 0.05.

Transcriptome data from SK-Mel-29 cells with TAp73α or DNp73β overexpression (Figure 3) were used to verify the p73-coregulator interactions. Differential expression analysis identified 4540 upregulated and 5017 downregulated genes upon TAp73α, as well as 4578 upregulated and 5898 downregulated genes following DNp73β expression (FC ≥ 1.5 or ≤−1.5, *p* < 0.05). Comparison with 564 unique coregulators revealed that 94 are induced and 87 repressed by TAp73α (Figure 8A, left), while 80 are induced through DNp73β and 86 repressed (Figure 8B, left), demonstrating that these isoforms regulate different subsets of coTFs. Since the survival-associated cofactors did not show consistent expression trends in the patient data (Figure 7), we examined their isoform-specific regulation. We found that TAp73α upregulates *IRF9* and *PROX1*, both associated with patient survival, while repressing adverse prognostic factors *MYBL2* and *HOXC9*. Contrariwise, DNp73β induces *FOXF2* and *MYBL2* but suppresses *SATB1*, *STAT1*, and *IRF9*, suggesting a shift toward unfavorable outcome. Remarkably, *MYBL2* and *IRF9* were oppositely regulated by TA and DN, suggesting their role as potential p73-dependent regulatory switches.

In addition, we analyzed TA- and DN-specific protein interactions using CoIP-MS [2,5]. Since transcriptional regulation requires nuclear localization, interactors were filtered by subcellular compartment (Figure 8C). Although the majority of proteins were assigned to mitochondria, plasma membrane/extracellular region, cytoplasm, Golgi, or ER, 22% of identified cofactors were localized to the nucleus, highlighting the functional diversity of p73 beyond transcriptional regulation. Consequently, the nuclear interactors detected by CoIP-MS represent a complementary set to the coTFs predicted from JASPAR. Importantly, PATZ1 consistently emerged as a DNp73β-associated factor across all datasets, underscoring its potential functional relevance, whereas the apparent non-overlap reflects the diverse mechanisms through which p73 cofactors operate, including transient or chromatin-proximal regulatory effects that may not be captured by CoIP-MS.

To expand coverage beyond JASPAR, we extended the analysis to the TFDB resource [21], which distinguishes DNA-binding transcription factors (TFDB-TFs; n = 1638) from non-DNA-binding cofactors (TFDB-CoFs; n = 1025). Over 500 JASPAR entries matched TFDB-TFs, while 60 were found only in JASPAR (Figure 8D,E). In this comprehensive framework, p73 itself was the only common interactor for both isoforms and PATZ1 was again identified as DN-specific candidate, thus consistently found in JASPAR, TFDB-TF, and CoIP-MS. Additionally, the analysis highlighted several bona fide DNA-binding TFs, including SUB1 and YBX3 with TAp73α (Figure 8D) and AHCTF1 and HMGB3 with DNp73β (Figure 8E), which currently lack JASPAR motifs and represent promising candidates for future CUT&RUN validation. The strongest enrichment was observed when CoIP-MS interactors were matched with TFDB cofactors, identifying 30 for TAp73α and 7 for DNp73β. This suggests a broader and more modular cofactor network for TAp73α. Among these, HDAC2 which binds to TAp73 [5] supports the role of chromatin-modifying cofactors in TA-dependent regulation. Overall, TAp73α displayed a higher number of cofactor interactions than DNp73β, indicating greater dependence on chromatin-associated mechanisms. While no isoform-specific coTFs were found for p73, PATZ1 proved to be a robust DNp73β interactor associated with poor survival rates and significantly upregulated in melanoma patients (Figure 8F,G).

## 4. Discussion

In this study, we present an integrative analysis of the genome-wide binding profiles and regulatory programs of TAp73 and DNp73. Although both isoforms bind largely overlapping genomic regions due to their shared DNA-binding domain, they differ significantly in terms of transcriptional outcome and coTF recruitment. This underscores that their functional divergence is determined less by DNA binding itself than by differences in the coregulator context and promoter architecture. Building on our previous work, which characterized isoform-specific effects on gene expression and protein interactions for individual candidates, we expanded these findings by integrating CUT&RUN chromatin profiling for each isoform. This enables the identification of genome-wide isoform-dependent coTFs and cofactors that influence p73 binding and the assessment of their enrichment or depletion in promoter regions associated with cancer-related gene regulation.

The DNA-binding affinity of p73 has been well studied in vitro and modeled as a position-specific scoring matrix (PSSM) in the JASPAR database [9]. These theoretical genome-wide binding motifs formed the starting point of our analysis. We considered (a) the predicted p73 motifs and (b) motifs of other transcription factors in JASPAR as potential coregulators that may promote or hinder p73 binding. The CUT&RUN data were used to assess whether predicted binding motifs were enriched or depleted. This approach identifies motifs that are enriched (supportive) or depleted (adverse) in proximity to p73 sites, irrespective of genomic context.

The transcription factor database JASPAR provides an experimentally derived and in vitro optimized motif [9] for the DNA-binding domain of p73, which is free of biological interference. By using these motifs as reference, we avoided the uncertainty of peak-based identification in CUT&RUN data. This justified the minimal confirmation threshold of a single read and enabled consistent analysis across both overexpression and physiological (control) conditions, despite differences in sequencing coverage. As a result, a p73 motif is expected roughly every 1000 base pairs. Because of this high density, nearly all well-defined consensus peaks of the CUT&RUN data present a p73 motif. The proportion of predicted sites confirmed is roughly double what would be expected based on genome-wide CUT&RUN coverage. This indicates that even regions of low coverage are not random and still support a nuanced analysis of isoform-specific differences in the CUT&RUN data.

The most reliable peak recognition and strongest signals were observed near transcription start sites, indicating that the cofactors’ functional interpretation was best guided by association with promoter regions. For the coTF analysis, we assessed p73 binding sites genome-wide, benefitting from a ~2000-fold increase in site numbers and improving the statistical power. Within a 100 bp neighborhood, across all four million predicted binding sites, or within subsets such as the 500 bp upstream of transcription start sites as putative promoter regions, we identified other motifs whose presence increased (enriched) or decreased (depleted) the likelihood that a predicted p73 site was confirmed in CUT&RUN. The implementation is novel, though conceptually related to the approach by Rubin et al. [22].

A central finding was the consistent occurrence of PATZ1 as a DNp73β-specific factor fulfilling three independent criteria: (i) annotation as a DNA-binding TF in TFDB, (ii) presence of a defined JASPAR motif enriched near p73 binding sites, and (iii) direct interaction with DNp73β in CoIP-MS. Its association with poor survival rates in melanoma patients underscores its clinical relevance and positions it as a potential biomarker for isoform-driven tumor aggressiveness. The isoform-specific engagement with EMT-associated TFs is of particular interest. DNp73β acts primarily as an oncogene due to its binding profile, as it co-localized with other motifs of EMT inducers (e.g., PATZ1, AP-1, IRF2, PAX9, and RARA), providing a mechanistic link to its pro-invasive phenotype. This supports earlier findings that DNp73 enhances migration and metastasis via activation of SNAI2 and repression of E-cadherin [8]. Conversely, TAp73α displays dual roles and is engaged in the regulation of both tumor-suppressive and tumor-promoting gene programs. The recruitment of potential coTFs such as IRF2, IRF9, NFKB2, MYBL2, FOXF2, and REST not only supports EMT- but also inflammation-related transcriptional reprogramming by TAp73. The complexity of coregulator-mediated p73 functionality is further supported by our observation that Sp1 is a central co-binding partner for both isoforms, with nearly half of all CUT&RUN-confirmed p73 sites containing an SP1 motif. This is consistent with previous reports of Sp1 forming functional complexes with p73 to regulate genes such as *PUMA* and *Cyclin B1* [23,24]. Additional enriched motifs included those of JUNB, ATF7, PLAGL2, TFAP2C, and E2F1, transcription factors involved in proliferation, apoptosis, metastasis and lineage specification [25,26,27,28]. In particular, a cooperation with E2F1, which is well known to induce pro-metastatic and pro-inflammatory signaling [29], further suggests that TAp73 can promote EMT and immunomodulation in certain contexts. In this regard, *MYBL2* and *IRF9* emerged as oppositely regulated by the two isoforms, emphasizing their role as candidate p73-dependent regulatory switches that can tip the balance between tumor suppression and progression. These findings underpin the concept that isoform-dependent cofactor usage introduces a new regulatory layer to the molecular pathology of p73 in cancer and could be exploited for patient stratification.

This functional versatility can be explained by two complementary mechanisms: First, structured regions, particularly the TA-specific SAM domain, enable TAp73α to bind partners such as HDAC2, modulate transcriptional machineries like the REST-HDAC2 repressor complex, and facilitate processes that favor metastasis [5]. Moreover, the observed enrichment of REST motifs near confirmed TAp73α-binding sites suggests a spatial organization that facilitates their interaction and supports a genome-wide regulatory role for the previously proposed and experimentally validated mechanism. In contrast, DNp73β lacks the TA domain, does not interact with HDAC2, and forms less stable PPIs, as shown by our CoIP-MS data, underscoring the structural basis of isoform-specific interactions. Second, intrinsically disordered regions (IDRs) provide an additional level of regulatory flexibility. These regions facilitate dynamic multi-partner assemblies even in the absence of a structured transactivation module, allowing DNp73β to form regulatory complexes despite its truncated architecture. Such “fuzzy” interactions, mediated by weak and transient contacts that are not constrained by motif distances, are difficult to detect and model, but offer therapeutic opportunities [30,31]. Molecular glues or peptide mimetics could selectively stabilize or disrupt isoform-specific IDR-driven assemblies, introducing a viable route for the pharmacological modulation of transcriptional programs once considered undruggable [32,33].

A further interesting aspect of the PATZ1 DNp73β interaction is its role as a multifunctional regulator of signal transduction by the p53 family. It can interact with wild-type p53, bind to promoters of canonical p53 target genes such as *BAX*, *CDKN1A*, and *MDM2*, and enhance their expression, thereby promoting p53-mediated apoptosis in certain contexts. However, in cells lacking functional p53, PATZ1 can counteract these target gene effects and confer a survival advantage to the cells [34]. This context-dependent transcriptomic duality, together with the suppressive activity of DNp73, mediated by competition for DNA binding with p53 and the formation of inactive heterooligomers [4], underpins the role of PATZ1 as a potent modulator of the activity of members of the p53 family. Moreover, our observation that PATZ1 is specifically associated with DN suggests that this isoform utilizes PATZ1 to drive EMT programs, compensating for the lack of an intrinsic transactivation domain.

Our integrative approach also addresses an important conceptual issue: the prognostic significance of coregulators cannot be deduced from expression data alone [35]. For example, *MEF2B* was associated with a favorable patient outcome even though it was downregulated in the corresponding subgroup. Such discrepancies illustrate that survival dependencies do not result from expression levels per se, but from the regulatory context, including promoter-specific interactions, network integration, and the interplay between activating and repressive partners. From this perspective, motif- and binding-centered signatures provide a more accurate assessment of functional dependencies than bulk transcriptomics alone and lend greater explanatory depth to clinical observations.

Our workflow is in line with the requirements for modern cancer therapies that are tailored to tumor heterogeneity and increasingly guided by integrative and multiomic-based approaches [36]. Advances in high-throughput transcriptomics and machine learning are propelling the shift toward precision oncology [35,37,38]. Next-generation sequencing has revolutionized the ability to capture molecular and epigenetic information, but is often insufficient on its own to annotate the functions of genes embedded in complex signaling pathways [35]. Integrative frameworks that combine sequencing data with motif-based bioinformatics and expression profiling are essential for uncovering regulatory dependencies and identifying clinically applicable biomarkers [39,40]. Our study demonstrates this principle by showing how isoform-specific coregulator networks of TA and DN can be systematically resolved, providing comprehensive information about their distinct roles in EMT, immune signaling, and metastasis, and could link these networks to patient treatment outcomes.

Given the potential translational implications of our findings, it is important to consider methodological factors that could influence data interpretation. Although overexpression of a separate isoform enabled robust CUT&RUN profiling and provided highly reliable binding data, it is essential to interpret the results in the context of physiological expression levels. Here, promoter-centric enrichment, consistency with histone mark controls, and validation through JASPAR-supported motifs strengthen data reliability. Potential discrepancies between motif predictions, expression patterns, and survival associations reflect the multifactorial nature of transcriptional regulation and the incompleteness of current databases. Furthermore, clinical bulk sequencing data may obscure cell type-specific dynamics. Future efforts should therefore also focus on profiling endogenous isoforms with single-cell and spatial resolution, as well as expanding motif libraries to include non-canonical interactors.

The JASPAR database aggregates binding profiles from different species [9], whereby in many cases the motifs of human and mouse orthologs are virtually indistinguishable. To avoid redundancies, we have focused primarily on human factors. However, data from other species can provide valuable insights when no defined binding profile is available for the human ortholog, highlighting additional TFs as potential modulators of p73-driven processes. Since p73 is evolutionarily ancient and existed before the well-known p53 [41], binding data from orthologous TFs can be particularly revealing. Interestingly, some plant-derived motifs exhibit short, GC-rich architectures that resembles CpG sites. Although unlikely to represent direct human counterparts, this could still be relevant for isoform-specific DNA binding. Looking ahead, the inclusion of motifs from orthologs, coTFs without direct human counterparts, and de novo patterns derived from strong CUT&RUN binding sites could further refine regulatory profiles [42] and help uncover new PPI partners that shape p73-driven transcriptional programs. Moreover, we anticipate that our findings will encourage a broad range of follow-up experiments. As CUT&RUN profiles for the newly identified p73-coTFs become available, their co-occurrence with the TF at predicted binding sites can be further systematically quantified, transforming current statistical associations into experimentally validated interactions. Targeted reporter assays can be employed to confirm the role of individual cofactors at specific loci, while the uniform use of log ratios across all enrichment analyses ensures direct comparability, thereby providing a robust foundation for subsequent studies.

## 5. Conclusions

This work provides a novel integrative framework for analyzing isoform-specific p73 regulatory networks. By combining CUT&RUN, JASPAR motif prediction, transcriptomics, and proteomics, we demonstrate how different coregulator repertoires underlie the divergent functions of TA and DN in, for instance, EMT and immune signaling. The identification of PATZ1 as a novel DNp73β interactor illustrates how such multilayered strategies can uncover clinically relevant vulnerabilities that escape conventional analyses. At the same time, the dual role of TAp73 emphasizes the need for therapeutic strategies that consider isoform-specific functions rather than assuming predefined tumor-suppressive or oncogenic identities. Integrating these approaches into cancer patient stratification could ultimately pave the way for precise clinical strategies that exploit p73-specific vulnerabilities for therapy.

## Data Availability

The datasets generated in this study are available upon request or can be found in online repositories: Affymetrix Clariom D transcriptome data (E-MTAB-14704) and CUT&RUN sequencing data (E-MTAB-15709) via https://www.ebi.ac.uk/arrayexpress (accessed on 21 July 2025), Mass spectrometry raw dataset on https://www.ebi.ac.uk/pride (PXD058816) (accessed on 21 July 2025). All code that contributed to the analysis is made available at https://github.com/IEGT/jaspar-mapping (accessed on 21 July 2025).

## Supplementary Materials

The following supporting information can be downloaded at: https://www.mdpi.com/article/10.3390/biom16010063/s1, Table S1: Densitometric values of the β-Actin bands; Table S2: Densitometric values of the p73 bands, and Original Western Blot images.
