# Supplementary figures and images for: Integrative Multimodal Profiling of TAp73 and DNp73 Reveals Isoform-Specific Transcriptomic Coregulator Landscapes in Cancer Programs
Source: Biomolecules. 2025 Dec 31;16(1):63. doi: 10.3390/biom16010063 (PMC12839168; doi:10.3390/biom16010063)

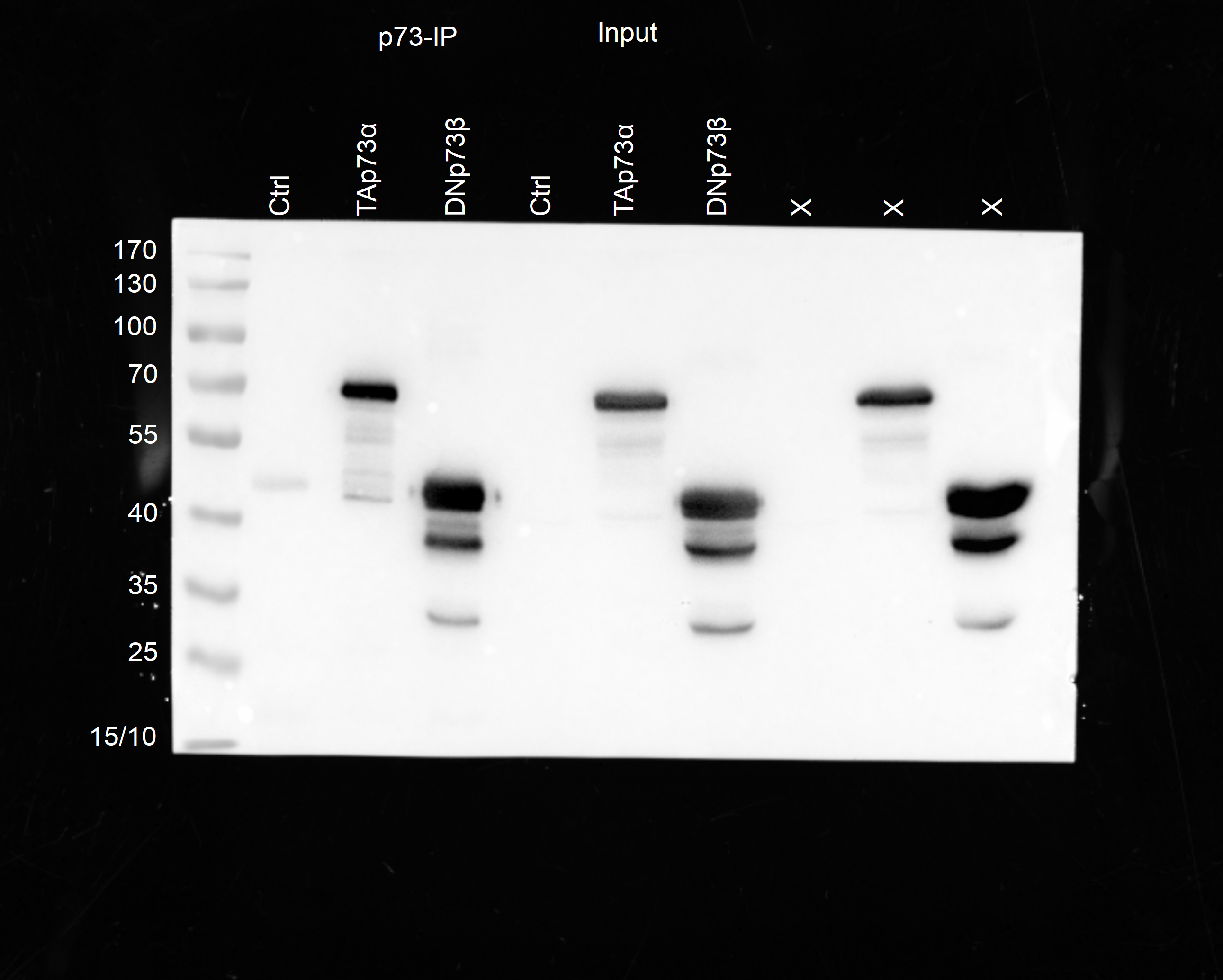

Supplement: Supplementary file 1 [file biomolecules-16-00063-s001.zip › biomolecules-4040752-supplementary/p73 Raw.tif]

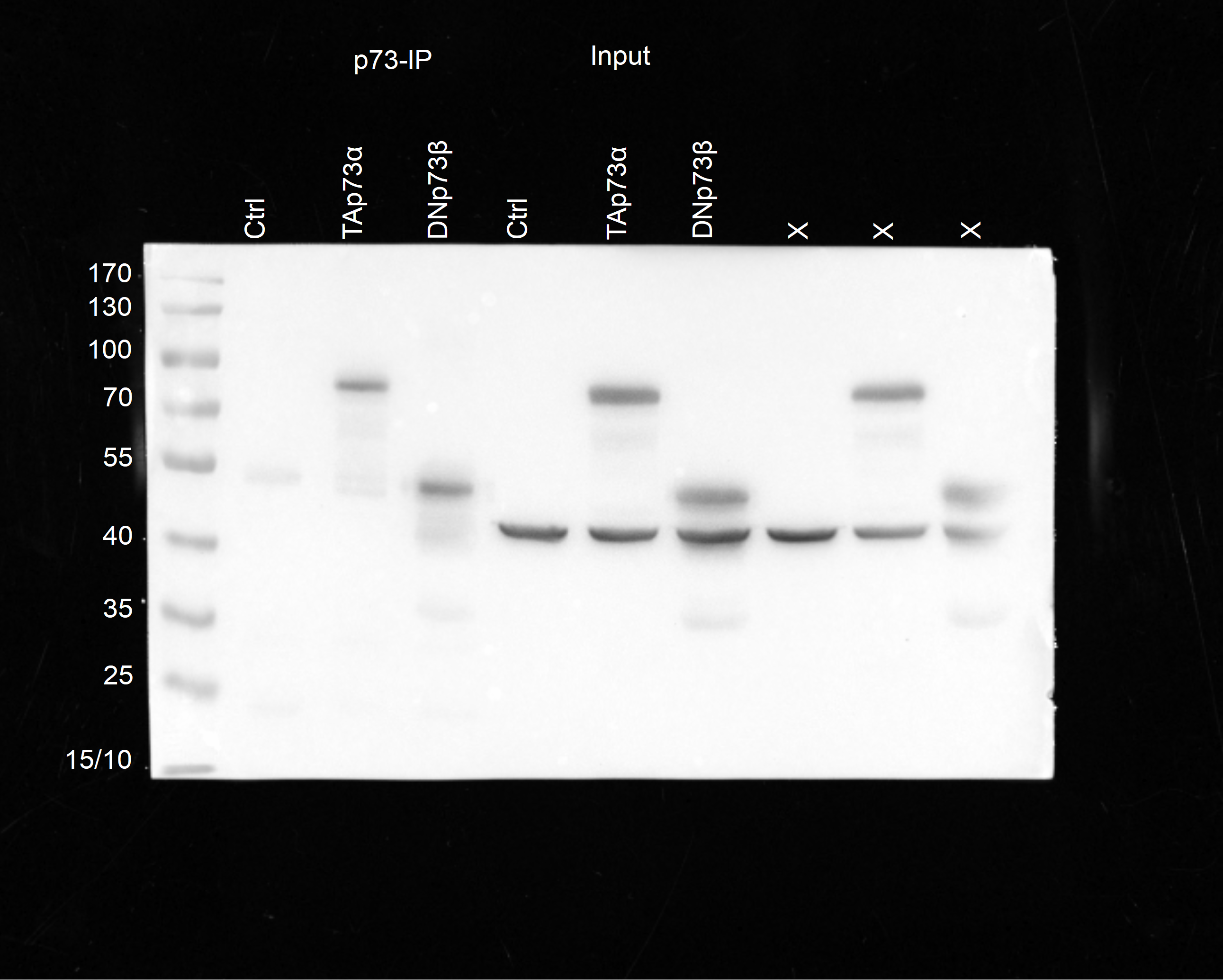

Supplement: Supplementary file 1 [file biomolecules-16-00063-s001.zip › biomolecules-4040752-supplementary/ß-Actin Raw.tif]
